# Spinal deformity progression after posterior segmental instrumentation and fusion for idiopathic scoliosis

**DOI:** 10.1007/s11832-015-0632-5

**Published:** 2015-01-20

**Authors:** Vidyadhar V. Upasani, Daniel J. Hedequist, M. Timothy Hresko, Lawrence I. Karlin, John B. Emans, Michael P. Glotzbecker

**Affiliations:** 1 Department of Orthopedic Surgery, Boston Children’s Hospital, Boston, MA USA; 2 Rady Children’s Hospital San Diego, 3030 Children’s Way, Suite 410, San Diego, CA 92123 USA

**Keywords:** Spinal deformity, Spinal deformity progression, Posterior segmental instrumentation and fusion, Idiopathic scoliosis

## Abstract

**Study design:**

Retrospective chart and radiographic review.

**Purpose:**

To assess the incidence of and variables associated with spinal deformity progression after posterior segmental instrumentation and fusion at a single institution. Progression of the scoliotic deformity after posterior instrumented spinal fusion has been described. Recent studies have concluded that segmental pedicle screw constructs are better able to control deformity progression.

**Methods:**

Retrospective review of a consecutive series of idiopathic scoliosis patients (*n* = 89) with major thoracic curves (Lenke types 1–4) treated with posterior segmental instrumentation and fusion. Deformity progression was defined as a 10° increase in Cobb angle between the first-erect and 2-year post-operative radiographs. Clinical and radiographic data between the two cohorts (deformity progression versus stable) were analyzed to determine the variables associated with deformity progression.

**Results:**

Patients in the deformity progression group (*n* = 13) tended to be younger (median 13.7 vs. 14.7 years) and experienced a significant change in height (*p* = 0.01) during the post-operative period compared to the stable group (*n* = 76). At 2-years post-op, the patients in the deformity progression group had experienced a significantly greater change in upper instrumented vertebra (UIV) angulation, lower instrumented vertebra (LIV) angulation, and apical vertebral translation (AVT). Two-year post-op Scoliosis Research Society questionnaire (SRS-22) scores in the appearance domain were also significantly worse in the deformity progression group. Patients in the deformity progression group had a significantly greater difference between the lowest instrumented vertebra and stable vertebra compared to patients in the stable group (*p* = 0.001).

**Conclusions:**

Deformity progression after posterior spinal fusion does occur after modern segmental instrumentation. Segmental pedicle screw constructs do not prevent deformity progression. Skeletally immature patients with a significant growth potential are at the highest risk for deformity progression. In immature patients, extending the fusion distally to the stable vertebra may minimize deformity progression.

**Level of evidence:**

Level III.

## Introduction

The primary goals in the surgical treatment of adolescent idiopathic scoliosis (AIS) are to achieve a well-balanced spine, arrest deformity progression, and maintain correction by achieving a solid arthrodesis. However, progression of the scoliotic deformity after posterior spinal fusion has been described [1–4]. Various etiologies for loss of correction have been proposed, including pseudarthrosis, implant failure, incorrect selection of fusion levels, “adding-on” [5], biologic plasticity of the fusion mass [6, 7], and the crankshaft phenomenon (continued anterior growth of the spine) [8].

Recent studies have proposed that pedicle screws are better able to control the three columns of the spine and may decrease the incidence of deformity progression after posterior spinal instrumentation and fusion [9, 10]. In our anecdotal experience, this has not been the case. Therefore, the purpose of this study was two-fold: (1) to assess the incidence of spinal deformity progression after posterior segmental instrumentation and fusion in the treatment of idiopathic scoliosis at a single institution, and (2) to analyze the variables associated with deformity progression in this patient population.

## Materials and methods

After obtaining institutional review board approval, a retrospective review of a consecutive series of idiopathic scoliosis patients treated at a single institution was performed. All patients with major thoracic curves (Lenke types 1–4) treated with posterior segmental instrumentation were included if they had minimum 2-year clinical and radiographic follow-up. Exclusion criteria included anterior spinal release or instrumentation and fusion, and incomplete radiographic or clinical data. Data were collected from the pre-operative, immediate post-operative (4–6 weeks post-op), 1-year post-operative, and 2-year post-operative visits. A total of 402 idiopathic scoliosis patients were surgically treated at our institution between November 2003 and August 2008. Of these, 285 gave their consent to enter the prospective patient database that was used to analyze the current cohort, and 230 of the 285 patients (80 %) had at least 2-year post-operative follow-up. Eighty-nine patients were included in this analysis based on our inclusion criteria of main thoracic scoliosis (Lenke types 1–4) and posterior-only segmental instrumentation. Segmental instrumentation was defined as more than 80 % of fixation points were instrumented. Thirty-three percent of patients had all pedicle screw constructs, and 67 % of patients had hybrid constructs with hooks, sublamina wires, and pedicle screws.

Although the instrumentation type differed, similar techniques were used to perform the deformity correction and to achieve a solid arthrodesis. Complete facetectomies were performed at each instrumented level. Deformity correction was achieved using compression techniques on the convexity and distraction on the concavity, as well as in situ bending. En bloc derotation maneuvers were used in patients treated with pedicle screws. Thorough decortication of the posterior elements was performed at the end of the procedure and local autograft as well as cancellous allograft bone chips were used in the fusion mass.

Clinical measures included height, weight, and inclinometer measurements (proximal thoracic, thoracic, and thoracolumbar). Height measurements were performed at each clinic visit using an Ayrton Stadiometer Model S100 (Prior Lake, MN), with a reported precision of 0.15 cm. Radiographic parameters included proximal thoracic, main thoracic, and thoracolumbar coronal Cobb angles, thoracic and thoracolumbar sagittal Cobb angles, apical vertebral translation (AVT), coronal and sagittal balance, T1 tilt, Risser grade, and state of the triradiate cartilage (TRC). We also determined the implant density for each patient, as well as the percent of fixation sites instrumented with pedicle screws. Subjective scores were collected at each clinic visit using the Scoliosis Research Society questionnaire (SRS-22). Based on previous studies, deformity progression was defined as a 10° increase in the major coronal Cobb angle between the first post-operative and 2-year post-operative radiographs [11–13]. Selection of fusion levels was also assessed in these patients. For each patient, the stable vertebra (SV) (most distal vertebra in the major curve that is bisected by the center sacral vertical line), neutral vertebra (NV) (most distal vertebra in the major curve that is neutrally rotated based on the symmetric appearance of the pedicles), touch vertebra (TV) (last vertebra with the pedicle touched by the center sacral vertical line), and the last instrumented vertebra (LIV) were recorded.

### Statistical analysis

Clinical and radiographic data between the two groups [deformity progression versus no progression after instrumented fusion (“stable”)] were analyzed to determine the variables associated with deformity progression. Patient and curve characteristics were summarized pre-operatively, at first erect, at 1-year follow-up, and at 2-year follow-up for all subjects and between deformity progression and control subjects. Continuous characteristics were compared using univariate logistic regression and ordinal characteristics were compared using the Cochran–Armitage test for trend or a Mann–Whitney *U*-test. Change in measurement over time was analyzed between deformity groups using mixed model analysis with a compound symmetry correlation structure. Univariable and multivariable logistic regression was used to assess pre-operative and post-operative risk factors of deformity progression. All tests were two-sided and *p*-values less than 0.05 were considered significant.

## Results

**Fig. 1:**
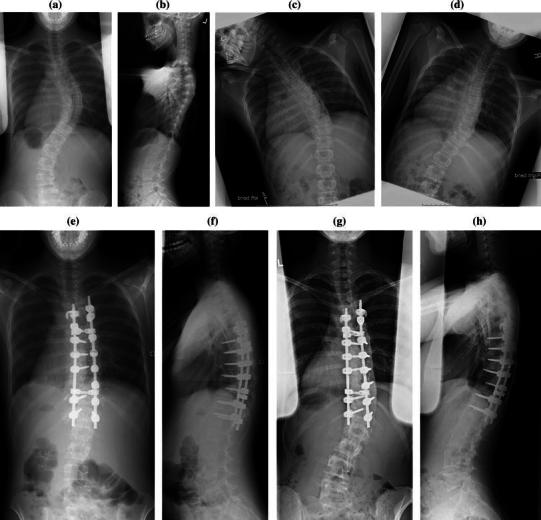
A 12 years and 8 months old, Risser grade 0, pre-menarchal female with Lenke type 1B idiopathic scoliosis. Pre-operative posterior-anterior (PA) (**a**), lateral (**b**), *left* bend (**c**), and *right* bend (**d**) radiographs demonstrate a 57° main thoracic curve. First-erect post-operative PA (**e**) and lateral (**f**), and 5-year post-operative PA (**g**) and lateral (**h**) radiographs demonstrate correction of the coronal plane deformity down to 15° with a T5–T12 posterior instrumented spinal fusion. The patient was fused to one level short of the stable vertebra. The deformity then progressed to 41° at final follow-up. At the most recent follow-up, the patient has developed a 16° thoracic rib prominence with worsening trunk shift to the right and waist asymmetry

Seventy-six patients (86 %) were in the stable control group and 13 patients (14 %) were found to experience deformity progression of more than 10° in the coronal plane within 2 years of surgery (Fig. 1). The stable group comprised 14 boys and 62 girls, and the deformity progression group comprised three boys and ten girls. The average follow-up for this cohort was 4.2 years (range 2.0–8.3 years).

### Clinical measures

Patients in the deformity progression group tended to be younger based on chronologic age. The average age in the deformity progression group was 13.7 years and in the stable group it was 14.7 years (*p* = 0.14). The 14 boys in the stable group had a median age of 15.1 years and the 62 girls in the stable group had a median age of 14.2 years. The three boys in the deformity progression group had a median age of 13.7 years and the ten girls in the deformity progression group had a median age of 12.6 years.

There was a significant difference in the change in height from 1-year follow-up to 2-year follow-up with respect to deformity progression. The average change in height for the deformity progression group was 2.6 cm, compared to 0.8 cm for the stable group (*p* = 0.01). There was also a significant difference in the change in height from pre-operative to 2-year follow-up with respect to deformity progression. The average change in height for the deformity progression group was 6.4 cm, compared to 3.5 cm for the stable group (*p* = 0.003). There was no association between weight or inclinometer measurements and deformity progression.

Two patients in the stable group required revision procedures for late infection. The implants were removed at 2.5 and 3 years post-op, respectively. They were followed for at least 1 year after the revision surgery, with no significant increase in the spinal deformity at the final follow-up. Two patients in the deformity progression group also required a revision procedure. One patient underwent removal of implants at 3 years post-op for late infection after her deformity had already progressed. At the final follow-up, there was no significant increase in her radiographic deformity. A second patient required an irrigation and debridement on post-operative day 10 for persistent wound drainage. He completed a course of intravenous antibiotics and his implants were retained. Additionally, one patient in the deformity progression group was found to have a broken screw at 1.5 years post-op. This was asymptomatic and did not require a revision procedure. Further evaluation with a computed tomography scan and nuclear bone scan over the next year did not demonstrate pseudarthrosis or additional implant failure.

### Radiographic measures

Tables 1, 2, 3, and 4 present radiographic data from the pre-operative, first-erect post-operative, 1-year post-operative, and 2-year post-operative visits, respectively. There was no significant relationship between menarchal status, TRC, Lenke classification, T1 tilt, or coronal balance and deformity progression. While more than 50 % of the patients in the deformity progression group were Risser grade 0 or 1, the distribution of the Risser grades between the two groups was not statistically significant (Table 5). The mean change in the major coronal Cobb angle was 1.7° in the stable group (pre-op: 58.5°; post-op: 22.1°; 2-year post-op: 23.8°) versus 14.7° in the deformity progression group (pre-op: 52.9°; post-op: 17.5°; 2-year post-op: 32.2°).

**Table 1:** Pre-operative clinical and radiographic data

Variable	All subjects (*N* = 89)		Deformity progression (*n* = 13)		Control (*n* = 76)		*p*-Value
	Mean	±SD	Mean	±SD	Mean	±SD	
Age (years)	14.5	±2.19	13.7	±1.87	14.7	±2.22	0.14
Height (cm)	160.1	±8.84	161.3	±11.86	159.9	±8.29	0.59
Weight (cm)	54.7	±11.45	52.1	±12.06	55.2	±11.36	0.37
Proximal inclinometer [median (IQR)]	4	(2–6)	5	(0–3)	4	(2–6)	0.16
Thoracic inclinometer [median (IQR)]	15	(12–15)	12.5	(12–14)	15	(12–15)	0.49
Thoracolumbar inclinometer [median (IQR)]	6	(4–7)	7	(2–7)	6	(4–7)	0.95
Touch vertebrae [median (IQR)]	22	(20–22)	22	(22–23)	22	(20–21)	0.11
Cobb proximal thoracic	27.6	±9.71	26.7	±9.17	27.8	±9.85	0.70
Cobb main thoracic	57.7	±11.81	52.9	±7.78	58.5	±12.22	0.12
Cobb thoracolumbar	36.1	±11.72	33.2	±12.40	36.6	±11.61	0.32
AVT proximal	4.1	5.34	5	4.92	3.9	5.43	0.39
AVT main	48.4	±15.18	43.2	±10.14	49.2	±15.77	0.19
AVT lumbar	16.7	±12.71	15.4	±12.26	16.9	±12.85	0.53
Coronal balance	15.7	±10.77	20.8	±13.30	14.8	±10.12	0.12
T1 tilt	3.8	±4.43	4	±3.67	3.8	±4.57	0.28

*SD* standard deviation; *IQR* interquartile range (25th percentile–75th percentile); *AVT* apical vertebral translation

**Table 2:** First-erect clinical and radiographic data

Variable	All subjects (*N* = 89)		Deformity progression (*n* = 13)		Control (*n* = 76)		*p*-Value
	Mean	±SD	Mean	±SD	Mean	±SD	
Time from surgery to first erect (days)	29.7	±13.77	27.1	±11.76	30.1	±14.10	0.45
Percent correction	57.3	±11.84	52.6	±7.76	58.2	±12.26	0.12
Cobb proximal thoracic	12.1	±8.42	10.8	±12.76	12.3	±7.53	0.54
Cobb main thoracic	21.4	±8.44	17.5	±6.69	22.1	±8.56	0.08
Cobb thoracolumbar	19.6	±10.08	18.4	±8.02	19.8	±10.42	0.64
AVT proximal	4.1	±4.92	6.3	±5.54	3.8	±4.74	0.19
AVT main	13.2	±8.53	9.5	±5.56	13.8	±8.82	0.20
AVT lumbar	17.3	±13.01	15.5	±14.68	17.6	±12.78	0.27
Coronal balance	16.2	±11.69	17.4	±15.79	16	±10.96	0.21
T1 tilt	4.6	±4.41	5.5	±5.97	4.4	±4.12	0.49
UIV tilt	11.7	±1.32	3.6	±8.0	3.1	±6.5	0.70
LIV tilt	21.2	±2.03	4.5	±8.5	6.1	±7.4	0.52

*SD* standard deviation; *AVT* apical vertebral translation; *UIV* upper instrumented vertebra; *LIV* lower instrumented vertebra

**Table 3:** One-year post-operative clinical and radiographic data

Variable	All subjects (*N* = 89)		Deformity progression (*n* = 13)		Control (*n* = 76)		*p*-Value
	Mean	±SD	Mean	±SD	Mean	±SD	
Time from surgery to 1-year follow-up (days)	369.6	±59.22	349.2	±79.65	373.2	±54.72	0.18
Height (cm)	164.2	±8.23	165	±11.71	164	±7.54	0.69
Weight (cm)	59.4	±13.45	55.4	±11.03	60.1	±13.79	0.24
Proximal inclinometer [median (IQR)]	2	(0–3)	2.5	(0–4)	2	(0–2)	0.18
Thoracic inclinometer [median (IQR)]	7	(5–7)	7	(4–7)	6.5	(5–7)	0.69
Thoracolumbar inclinometer [median (IQR)]	0	(0–2)	0	(0–2)	1	(0–2)	0.84
Cobb proximal thoracic	13.9	±9.24	15.5	±9.89	13.7	±9.17	0.52
Cobb main thoracic	24.9	±8.55	26.6	±6.63	24.6	±8.85	0.43
Cobb thoracolumbar	19.4	±10.10	19.1	±9.33	19.4	±10.30	0.91
AVT proximal	4.3	±5.26	4.2	±3.72	4.3	±5.51	0.91
AVT main	16.8	±11.57	16.5	±8.57	16.8	±12.08	0.93
AVT lumbar	15.3	10.72	12.2	9.05	15.8	10.96	0.16
Coronal balance	13.7	±9.45	15.2	±12.86	13.5	±8.79	0.39
T1 tilt	4.8	±4.79	5.3	±4.97	4.7	±4.79	0.58
UIV tilt	7.4	±5.35	9.5	±4.03	7	±5.49	0.09
LIV tilt	8.8	±6.36	11.2	±6.07	8.4	±6.36	0.21

*SD* standard deviation; *IQR* interquartile range (25th percentile–75th percentile); *AVT* apical vertebral translation; *UIV* upper instrumented vertebra; *LIV* lower instrumented vertebra

**Table 4:** Two-year post-operative clinical and radiographic data

Variable	All subjects (*N* = 89)		Deformity progression (*n* = 13)		Control (*n* = 76)		*p*-Value
	Mean	±SD	Mean	±SD	Mean	±SD	
Time from surgery to 2-year follow-up (days)	780.4	±134.64	798.7	±123.31	777.4	±136.94	0.61
Height (cm)	164.4	±10.78	167.6	±12.98	163.9	±10.36	0.24
Weight (cm)	60.6	±14.34	58.1	±10.19	61.1	±14.95	0.49
Proximal inclinometer [median (IQR)]	1	(0–2)	0	(0–2)	2	(0–2)	0.54
Thoracic inclinometer [median (IQR)]	7	(4–7)	6.5	(5–8)	7	(4–7)	0.55
Thoracolumbar inclinometer [median (IQR)]	0	(0–2)	0	(0–2)	0	(0–2)	0.65
Cobb proximal thoracic	13.6	±8.29	15.5	±9.45	13.3	±8.10	0.39
Cobb main thoracic	25	±9.30	32.2	±7.32	23.8	±9.09	0.006
Cobb thoracolumbar	18.2	±10.15	21.9	±9.85	17.5	±10.13	0.15
AVT proximal	4.7	±5.63	6.8	±6.35	4.4	±5.46	0.21
AVT main	19.2	±11.82	22.2	±12.95	18.7	±11.63	0.28
AVT lumbar	14.3	10.5	12.8	9.99	14.5	10.63	0.44
Coronal balance	11.9	±9.51	12.8	±8.86	11.8	±9.66	0.04
T1 tilt	4.6	±4.75	6.9	±5.57	4.2	±4.52	0.20
UIV tilt	6.9	±6.01	11.6	±5.24	6.1	±5.79	0.02
LIV tilt	8.8	±5.90	13	±4.67	8.1	±5.82	0.04

*SD* standard deviation; *IQR* interquartile range (25th percentile–75th percentile); *AVT* apical vertebral translation; *UIV* upper instrumented vertebra; *LIV* lower instrumented vertebra

**Table 5:** Pre-operative and 2-year post-operative Risser grades

Variable	All subjects (*N* = 89)	Deformity progression (*n* = 13)	Control (*n* = 76)	*p*-Value
Pre-operative Risser grade				0.32
0	14 (16 %)	4 (31 %)	10 (13 %)	
1	17 (19 %)	3 (23 %)	14 (18 %)	
2	11 (12 %)	1 (8 %)	10 (13 %)	
3	15 (17 %)	2 (15 %)	13 (17 %)	
4	13 (15 %)	2 (15 %)	11 (15 %)	
5	19 (21 %)	1 (8 %)	18 (24 %)	
Two-year post-operative Risser grade				0.46
0	0 (0 %)	0 (0 %)	0 (0 %)	
1	5 (6 %)	0 (0 %)	5 (7 %)	
2	8 (9 %)	2 (15 %)	6 (8 %)	
3	6 (7 %)	1 (8 %)	5 (7 %)	
4	26 (29 %)	4 (31 %)	22 (28 %)	
5	44 (49 %)	6 (46 %)	38 (50 %)	

The mean absolute LIV angulation for the deformity progression group was 4.5 ± 8.5° at first erect and for the stable group, it was 6.1 ± 7.4° (*p* = 0.52). At 2-year follow-up, however, the mean absolute LIV angulation for the deformity progression group was 13.0 ± 4.7° and for the stable group, it was 8.1 ± 5.8° (*p* = 0.04). The mean change in LIV angulation from first erect to 2-year follow-up for the deformity progression group was 5.5 ± 4.4°, compared to 0.5 ± 4.7° for the stable group (*p* = 0.005).

The mean absolute upper instrumented vertebra (UIV) angulation for the deformity progression group was 3.6 ± 8.0° at first erect and for the stable group, it was 3.1 ± 6.5° (*p* = 0.70). At 2-year follow-up, however, the mean absolute UIV angulation for the deformity progression group was 11.6 ± 5.2° and for the stable group, it was 6.1 ± 5.8° (*p* = 0.02). The median change in UIV angulation from first erect to 2-year follow-up for the deformity progression group was 4.3 ± 5.8°, compared to 0.8 ± 5.3° for the stable group (*p* = 0.03).

The mean absolute main thoracic AVT for the deformity progression group was 9.5 ± 5.6 mm at first erect and for the stable group, it was 13.8 ± 8.8 mm (*p* = 0.20). At 2-year follow-up, there was also no significant difference detected. The mean absolute AVT for the deformity progression group was 22.2 ± 12.9 mm and for the stable group, it was 18.7 ± 11.6 mm (*p* = 0.28). There was, however, a significant median change in AVT in the deformity progression group from first erect to 2-year follow-up of 12.6 ± 11.3 mm, compared to 4.8 ± 9.3 mm for the stable group (*p* = 0.02).

Both the percent of fixation sites instrumented with pedicle screws and implant density were significantly different between the two groups. The percent of fixation sites instrumented with pedicle screws (71 %) was significantly greater in the deformity progression group compared to the stable group (47 %) (*p* = 0.004). Additionally, the mean implant density in the deformity progression group (1.79) was also significantly greater than the median implant density in the stable group (1.63) (*p* = 0.029).

Selection of fusion levels was also assessed in these patients with main thoracic curves (Table 6). Patients in the deformity progression group had a significantly greater difference between the lowest instrumented vertebra and stable vertebra compared to the patients in the stable group (*p* = 0.001). This was also true for the difference between the lowest instrumented vertebra and the touch vertebra between the two groups (*p* = 0.04). The difference between the LIV and the neutral vertebra however was not significantly different between the two groups (*p* = 0.43).

**Table 6:** Selection of fusion levels

Variable	All subjects (*N* = 89)		Deformity progression (*n* = 13)		Control (*n* = 76)		*p*-Value
	Mean	±SD	Mean	±SD	Mean	±SD	
LIV–SV	−0.3	±1.9	−1.8	±0.99	0	±1.9	0.001
LIV–NV	2.4	±2.22	1.9	±2.29	2.4	±2.21	0.43
LIV–TV	−0.4	±2.33	−1.7	±1.93	−0.2	±2.33	0.04

*SD* standard deviation; *LIV* lower instrumented vertebra, *SV* stable vertebra, *NV* neutral vertebra, *TV* vertebra last touched by the center sacral vertical line

### Subjective scores

Table 7 presents the SRS-22 scores from the pre-operative and 2-year post-operative visits. Subjects in the deformity progression group showed no significant change in the appearance domain score from pre-operative to 2-year follow-up (*p* = 0.221). However, subjects in the stable group showed a significant increase (improvement) in the appearance domain score, with a median pre-operative score of 3.4 ± 0.6 and a 2-year follow-up score of 4.4 ± 0.53 (*p* < 0.001). Subjects in the deformity progression group showed a significantly lower SRS-30 appearance domain score at 2-year follow-up than the stable group. The mean score at 2-year follow-up for the deformity progression group was 4.0 ± 0.66 and for the stable group, it was 4.4 ± 0.53 (*p* = 0.044).

**Table 7:** Pre-operative and 2-year post-operative Scoliosis Research Society questionnaire (SRS-22) scores

Variable	All subjects (*N* = 89)		Deformity progression (*n* = 13)		Control (*n* = 76)		*p*-Value
	Mean	±SD	Mean	±SD	Mean	±SD	
Pre-operative SRS-22 scores							
Pain	4.1	±0.63	4.2	±0.66	4.1	±0.63	0.88
Appearance	3.4	±0.64	3.5	±0.70	3.4	±0.63	0.86
Activity	4.2	±0.45	4.3	±0.56	4.2	±0.43	0.80
Mental	3.9	±0.67	3.8	±0.82	3.9	±0.65	0.43
Satisfaction	3.2	±1.52	3.1	±1.71	3.3	±1.49	0.67
Subtotal	3.9	±0.43	3.9	±0.57	3.9	±0.41	0.91
Total score	3.9	±0.42	3.9	±0.60	3.9	±0.39	0.87
Two-year post-operative SRS-22 scores							
Pain	4.4	±0.60	4.2	±0.65	4.4	±0.59	0.21
Appearance	4.3	±0.56	4	±0.66	4.4	±0.53	0.02
Activity	4.4	±0.35	4.3	±0.37	4.4	±0.35	0.21
Mental	4.2	±0.73	4	±0.51	4.2	±0.76	0.56
Satisfaction	4.4	±0.79	4.3	±0.94	4.4	±0.77	0.61
Subtotal	4.3	±0.43	4.1	±0.38	4.3	±0.43	0.09
Total score	4.3	±0.43	4.1	±0.41	4.4	±0.42	0.10

## Discussion

In 1973, Dubousset [8] first reported deformity progression after posterior spinal fusion in young patients with paralytic scoliosis. This was thought to occur as a result of continued anterior spinal growth and was termed “the crankshaft phenomenon”. Then, in 1989, Dubousset et al. [1] reviewed all idiopathic and paralytic cases fused before the age of 11 years at Texas Scottish Rite Hospital for Children and Miami Children’s Hospital between 1966 and 1984, and concluded that deformity progression was inevitable in patients with considerable remaining growth and recommended anterior fusion to achieve stable correction.

Since that time, several authors have attempted to determine the variables associated with deformity progression. Lee and Nachemson [3] evaluated this phenomenon in 63 consecutive patients with idiopathic scoliosis who were all Risser grade 0 at the time of surgery. They found that patients treated with hybrid constructs with chronologic age of 11 years or younger, especially with a skeletal age of 10 years or younger, were more likely to experience deformity progression. In this cohort, the average deformity progression was 9° in the coronal plane and 7° in the axial plane, as determined by the Perdriolle method [14]. Sanders et al. [13] performed a similar analysis in 43 patients and found that an open TRC and a younger chronologic age at the time of surgery were significantly predictive of the amount of deformity progression the patient experienced. Hamill et al. [12] also evaluated the state of the TRC and found that an open TRC alone did not indicate a high likelihood of deformity progression. A few years later, Sanders et al. [15] repeated the analysis in a different cohort of 43 patients and found that posterior spinal fusion performed before or during the peak height velocity was a strong predictor of the crankshaft phenomenon.

Our results confirmed the importance of chronologic age, as the patients in the deformity progression group tended to be younger (mean 13.7 vs. 14.7 years old). These patients also experienced a significant increase in height. Between 1 and 2 years post-op, the mean change in height for the deformity progression group was 2.6 ± 4.2 cm, compared to 0.8 ± 2.8 cm for the stable group (*p* = 0.01), indicating that they were closer to their peak height velocity at the time of surgery. As found in previous studies [9, 12], Risser grade and the state of the TRC were not significant variables in our analysis.

In terms of the amount of deformity progression after posterior fusion, several radiographic variables were significantly different between the two groups in this cohort. As expected, based on our definition of deformity progression, the residual scoliosis at 2 years post-op was statistically significant in the coronal plane, as measured by the main thoracic Cobb angle (mean 32.2° vs. 23.8°). Additionally, the change in UIV angulation, LIV angulation, and main thoracic AVT were all significantly greater in the deformity progression group. Axial plane deformity was difficult to assess on radiographs in this cohort. Pedicle screws interfered with our ability to use the Nash–Moe [16] and Perdriolle methods, and we had insufficient data on implant dimensions to use the position of the pedicle screws to determine vertebral rotation [17].

Recent studies have concluded that posterior segmental constructs are better able to control the crankshaft phenomenon and may mitigate the need for a combined anterior fusion [9]. Burton et al. [11] evaluated 18 Risser grade 0 patients with idiopathic scoliosis and found no evidence of crankshaft phenomenon in patients treated with segmental hook and screw constructs. Tao et al. [18] compared hybrid versus all pedicle screw constructs in 67 idiopathic scoliosis patients and found that radiographic measures of deformity progression were significantly better in the all pedicle screw group. Similarly, Sarlak et al. [19] reported on seven juvenile idiopathic patients treated with segmental pedicle screw constructs. With a minimum of 5 years follow-up, they reported some deformity progression in three patients; however, they felt that it was not enough to recommend combined anterior spinal fusion in this skeletally immature patient population.

Additionally, a recent study by Hwang et al. [9] identified a 12 % loss of coronal correction at 2-year follow-up that was not associated with infection, adding-on, or pseudarthrosis. They found that loss of correction was statistically associated with a larger Cobb magnitude, apical translation, and T1 tilt angle. The selection of fusion levels was not analyzed; however, patients with all pedicle screw constructs were found to have a lower rate of loss of correction compared to patients with hybrid fixation (10 vs. 20 %) [9].

In our study, a majority of the patients (67 %) were treated with a hybrid segmental construct with pedicle screws at the base and around the apex of the deformity, occasional sublamina wire fixation at the apex, and hooks at the top of the construct. Implant density was actually found to be significantly greater in the deformity progression group as compared to the stable group (mean 1.79 vs. 1.63). This may represent anticipation of post-operative deformity progression, and, therefore, surgeon bias to use a greater number of implants. Thirty-three percent of the patients were treated with segmental pedicle screw constructs with one or two hooks at the top. The percent of fixation sites instrumented with pedicle screws was also significantly greater in the deformity progression group.

The selection of fusion levels appeared to play a more important role in these patients. In general, main thoracic curves can be treated with selective or non-selective fusion based on the magnitude and flexibility of the compensatory curves. The lowest instrumented vertebra is typically selected by assessing the bend films, the position of the center sacral vertical line on the standing radiograph, as well as the end and neutral vertebrae of the main thoracic curve. Patients in this cohort who experienced deformity progression had a lowest instrumented vertebra which was nearly two motion segments cephalad to the stable vertebra (Table 6). In comparison, patients in the stable group were, on average, fused to the stable vertebra. Patients in both groups were fused about two vertebrae distal to the neutral vertebra.

While deformity progression was clearly evident in our patient cohort, it is difficult to differentiate the concepts of crankshaft phenomenon and adding-on below the LIV. Burton et al. [11] defined crankshaft as 10° of curve progression in the coronal Cobb angle or 10° of change in rib vertebral angle difference, and they defined adding-on as an increase in the scoliotic deformity outside the fused levels of 5° or more. Cho et al. [5] defined adding-on as an increase in Cobb angle of at least 5° and distalization of the end vertebra, or a change in disc angulation of 5° or greater below the LIV. Ultimately, a three-dimensional analysis of the fused segment will be required to clearly identify where the deformity progression occurred, either in the fused segment or over the adjacent levels.

The clinical significance of deformity progression remains unclear. In terms of subjective scores, only the appearance domain score was statistically different between the two groups at 2-years post-op (4.0 in the deformity progression group versus 4.4 in the stable group). Carreon et al. [20] recently published the minimum clinically important difference (MCID) for each domain of the SRS questionnaire and found the MCID of the appearance domain to be 0.98. Additionally, none of the 16 patients in this cohort required a revision surgery to correct the deformity that had worsened over the post-operative period. This finding likely represents a surgeon bias to avoid a complex revision procedure; however, none of the patients developed more than a 45° main thoracic coronal plane deformity.

This study has several limitations. Primarily, it was performed retrospectively and included only 24 % (98 out of 402) of the idiopathic scoliosis patients surgically treated at our institution during the determined time frame. Additionally, the sample size of the two groups was substantially different; however, we used appropriate statistical analyses to account for this difference. Additionally, a number of variables, including rod type and diameter, as well as surgical technique including derotation maneuvers, could not be controlled for in this analysis and could have biased our observation.

## Conclusion

It is clear from the current study that deformity progression can occur in growing children who undergo posterior spinal fusion for idiopathic scoliosis. What continues to be problematic is identifying patients at the greatest risk. The peak height velocity (PHV) has been identified as a risk factor; however, the PHV occurs prior to traditional determinants of growth remaining, such as Risser sign and bone age. Additionally, segmental pedicle screw constructs were not able to control deformity progression in this cohort, and the selection of fusion levels seemed to play a more important role, as patients in the deformity progression group were fused almost two levels cephalad to the stable vertebra.
